# ZBP1, an M1 Macrophage‐Associated Biomarker Identified by Machine Learning, Suppresses Tumorigenesis and Predicts Immunotherapy Response in Head and Neck Squamous Cell Carcinoma

**DOI:** 10.1111/jcmm.70953

**Published:** 2025-11-26

**Authors:** Feng Gao, Suya Wang, Tong Fang, Kaifang Wang, Ou Sha

**Affiliations:** ^1^ School of Dentistry, Institute of Stomatological Research, Medical School Shenzhen University Shenzhen China; ^2^ Medical School Shenzhen University Shenzhen China; ^3^ Cancer Centre, Faculty of Health Sciences University of Macau Macau China

**Keywords:** HNSCC, immunotherapy, machine learning, tumour microenvironment, ZBP1

## Abstract

Head and neck squamous cell carcinoma (HNSCC) is a highly aggressive cancer with restricted therapeutic options and unfavourable survival outcomes. To identify novel prognostic biomarkers and therapeutic targets, we investigated the role of macrophage polarisation in HNSCC progression. Using integrative computational approaches, including biological network algorithms and molecular subtyping, we established a robust gene signature associated with M1/M2 macrophage balance, which exhibited significant prognostic value in HNSCC patients. Further analysis employing multi‐model machine learning algorithms pinpointed ZBP1 as the pivotal gene, linking it to key clinical and immunological features, including disease progression, immune microenvironment remodelling, tumour mutational burden, and response to immune checkpoint inhibitors. Mechanistic studies confirmed ZBP1's tumour‐suppressive function, demonstrating its ability to inhibit HNSCC cell proliferation and migration in vitro. Moreover, macrophage co‐culture assays revealed that ZBP1 modulates immune regulation by restricting macrophage recruitment and altering polarisation dynamics. Collectively, our findings highlight ZBP1 as a promising prognostic biomarker and a potential immunotherapeutic target in HNSCC. This study not only enhances our understanding of macrophage‐mediated tumour immunity but also provides mechanistic insights into how ZBP1 integrates tumour‐intrinsic and immune‐regulatory pathways to influence HNSCC progression. These discoveries may contribute to the development of more precise therapeutic strategies for this aggressive malignancy.

## 1 Introduction

Head and neck squamous cell carcinoma (HNSCC) arises from squamous cells lining the mucosal surfaces of the head and neck region. As the sixth most common malignancy globally, HNSCC accounts for over 316,000 annual deaths, with major risk factors including tobacco use, alcohol consumption, human papillomavirus infection, and exposure to carcinogenic substances or radiation [1]. Standard HNSCC treatments consist of surgery, chemotherapy, radiation therapy, and targeted drugs. Early diagnosis and timely intervention are crucial for better patient outcomes. However, due to the generally unfavourable prognosis of HNSCC, developing new treatment strategies remains an urgent priority [2].

Macrophages are essential myeloid cells that serve vital functions in the innate immune system. These cells perform phagocytosis to eliminate pathogens while also processing external antigens for immune recognition and participating in the modulation of immune activity [3]. Macrophages undergo polarisation within invaded tissues in response to growth factors and cytokines. These immune cells are broadly classified into two major subtypes, M1 and M2 macrophages, which exhibit different surface markers, cytokine profiles, and biological functions. M1 macrophages primarily promote inflammation and enhance immune responses, while M2 macrophages generally exert anti‐inflammatory and immunosuppressive effects [4]. Macrophages exhibit high plasticity, allowing their polarisation state to shift in response to diverse signals from surrounding cells, tissues, and pathogens. This dynamic M1/M2 balance often reflects disease progression and may serve as a biomarker. Although macrophages have been widely proven to be associated with the oncogenesis of HNSCC [5, 6, 7], few studies have explored the M1/M2 macrophage ratio in HNSCC.

Immune checkpoint blockade (ICB) therapy has emerged as a promising approach in cancer immunotherapy, employing monoclonal antibodies targeting programmed cell death protein‐1 (PD‐1) or its ligand PD‐L1 [8]. However, clinical studies demonstrate that many patients exhibit poor responses to PD‐1/PD‐L1 inhibition, likely due to immunosuppressive mechanisms within the tumour microenvironment (TME) [9]. Tumour‐associated macrophages (TAMs) play a crucial role in mediating ICB resistance. Prostaglandin E2 (PGE2) activates the PI3K‐AKT‐mTOR pathway, suppressing T cell function while upregulating PD‐L1 expression [10]. Additionally, M2‐polarised TAMs enhance immune evasion by promoting PD‐L1 expression and dampening ICB efficacy [11, 12]. Given these findings, TAM‐targeted therapies may improve immunotherapy outcomes in HNSCC. We hypothesized that ZBP1, as a key mediator of M1 macrophage polarisation, drives antitumour immunity and predicts immunotherapy response in HNSCC.

In this study, we investigated macrophage polarisation dynamics by analysing M1/(M0 + M1 + M2) ratio‐associated genes and identified M.Cluster with significant prognostic value. Through rigorous machine learning screening, we identified ZBP1 as the most biologically significant gene within M.Cluster. Subsequent comprehensive analyses demonstrated that ZBP1 not only serves as a crucial biomarker with distinct mutation patterns but also plays critical roles in immune regulation and predicting immunotherapy response. Importantly, our findings establish ZBP1 as a key molecular nexus connecting macrophage biology with therapeutic outcomes in HNSCC, offering both mechanistic insights and translational applications for immunotherapy stratification.

## 2 Methods

### 2.1 Data Collection and Processing

Transcriptomic data analysis incorporated bulk RNA sequencing from TCGA‐HNSCC and microarray profiling (Illumina platform) from GSE65858 (GEO). For RNA‐seq data, expression quantification was performed using fragments per kilobase million (FPKM), followed by conversion to transcripts per million (TPM) values. Microarray datasets underwent preprocessing with Robust Multi‐array Average (RMA) normalisation.

### 2.2 WGCNA on M1/Macrophage

M0 macrophage, M1 macrophage, and M2 macrophage were calculated using the R package CIBERSORT [13]. The macrophage polarisation ratio (M1/Macrophage) was calculated as the proportion of M1 macrophages relative to the total macrophage population (M0 + M1 + M2). Using the WGCNA R package, we performed weighted gene co‐expression network analysis to systematically identify genes significantly correlated with the M1/Macrophage ratio [14]. Soft threshold settings were established to ensure a scale‐free topology network and generate a TOM matrix. A power of *β* = 4 and a scale‐free *R* ^2^ = 0.9 were used as the parameters. Magenta module genes were extracted for subsequent analysis.

### 2.3 Development of M.Cluster Based on Magenta Module Genes

Molecular subtyping analysis identified distinct patient clusters (M.Cluster) through consensus clustering using the Partitioning Around Medoids (PAM) algorithm based on magenta module gene expression. Gene expression patterns across M.Cluster subgroups were visualised using a heatmap. Kaplan–Meier survival curves were generated to compare clinical outcomes between clusters, and differentially expressed genes (DEGs) were visualised using volcano plots. Prognostically significant DEGs were identified through univariate Cox proportional hazards regression analysis.

### 2.4 Machine Learning for Dimension Reduction of Prognostic DEGs

We comprehensively identified prognostic biomarkers using multiple machine learning methods. The Least Absolute Shrinkage and Selection Operator (LASSO) regression selected key genes through L1 regularisation. CoxBoost analysis, a likelihood‐based boosting method for Cox models, further refined survival‐associated DEGs. Finally, Random Survival Forest (RSF), an extension of random forests for censored data, independently validated biomarker importance through ensemble tree‐based modelling. Prognostic feature selection was conducted through LASSO, CoxBoost, and RSF analysis, incorporating both transcriptional profiles and corresponding survival data.

### 2.5 Functional Annotation of ZBP1

To elucidate ZBP1's biological mechanisms and clinical implications, we conducted Gene Set Enrichment Analysis (GSEA) focusing on Gene Ontology (GO) functional terms to map its pathway associations [15]. Complementing this molecular characterisation, drug response prediction was performed using the oncoPredict R package, systematically evaluating ZBP1‐associated therapeutic vulnerabilities across standard chemotherapeutic agents [16]. The R package maftools was used to generate the mutation landscape [17].

### 2.6 Immune Characteristics of ZBP1

The immune infiltration landscape associated with ZBP1 expression was comprehensively analysed using four established computational approaches [18]. Immune cell abundance was evaluated through the following methodologies: Estimation of STromal and Immune cells in MAlignant Tumours using Expression data (ESTIMATE) algorithm quantified stromal and immune scores [19], Microenvironment Cell Populations‐counter (MCP‐counter) deconvolved specific immune cell subsets [20], Porpimol's analytical framework provided tumour microenvironment characterisation [21], and the Tumour Immune Estimation Resource (TIMER) platform systematically estimated immune infiltration levels [22]. This study further investigated the immunological associations of ZBP1 by systematically analysing its correlations with seven major immunomodulator categories [23].

### 2.7 In Vitro Validation on ZBP1

ZBP1 knockdown was performed using three specific siRNA sequences targeting human ZBP1 (NCBI Gene ID: 81030): si‐ZBP1‐#1 (GCAACATGCAGCTACAATTCC), si‐ZBP1‐#2 (GCAAAGTCAGCCTCAATTATT), and si‐ZBP1‐#3 (GCTGGATTTCCATTGCAAACT), with a non‐targeting siRNA (UUCUCCGAACGUGUCACGU) as a negative control. Cells were cultured under standard conditions: HSC‐3 and WSU‐HN30 in DMEM (Gibco, C11995500BT) supplemented with 10% FBS (Gibco, 10270106), and THP‐1 cells in RPMI‐1640 (Gibco, C11875500BT) with 10% FBS. For transfection, cells seeded in 6‐well plates were treated with 50 nM siRNA using Lipofectamine 3000 (Invitrogen, L3000015) following the manufacturer's protocol. After 48 h, RNA extraction was performed with TRIzol (Invitrogen, 15,596,026), followed by cDNA synthesis using HiScript III RT SuperMix (Vazyme, R323‐01). Quantitative PCR analysis was conducted with ChamQ SYBR qPCR Master Mix (Vazyme, Q711‐02) using ZBP1‐specific primers (forward: 5′‐GGACGATTTACCGCCCAGAT‐3′, reverse: 5′‐TCCAGCTGTTGGGTCCATTC‐3′) and GAPDH normalisation; si‐ZBP1‐#1 demonstrated optimal silencing efficiency and was selected for subsequent experiments. Cell proliferation was assessed via CCK‐8 assay (Dojindo, CK04), measuring absorbance at 450 nm after 24–72 h of incubation. Migration assays employed Transwell chambers (Corning, 3422) with serum‐free upper chambers and complete medium in lower chambers; after 24 h, migrated cells were fixed with 4% paraformaldehyde and stained with 0.1% crystal violet (Beyotime, C0121). For macrophage migration analysis, THP‐1 cells were differentiated into M0 macrophages using 100 ng/mL PMA (MedChemExpress, HY‐18739) for 24 h before co‐culture with transfected cancer cells in Transwell systems; migrated macrophages were quantified microscopically after staining.

## 3 Results

### 3.1 WGCNA on M1/Macrophage

**FIGURE 1:**
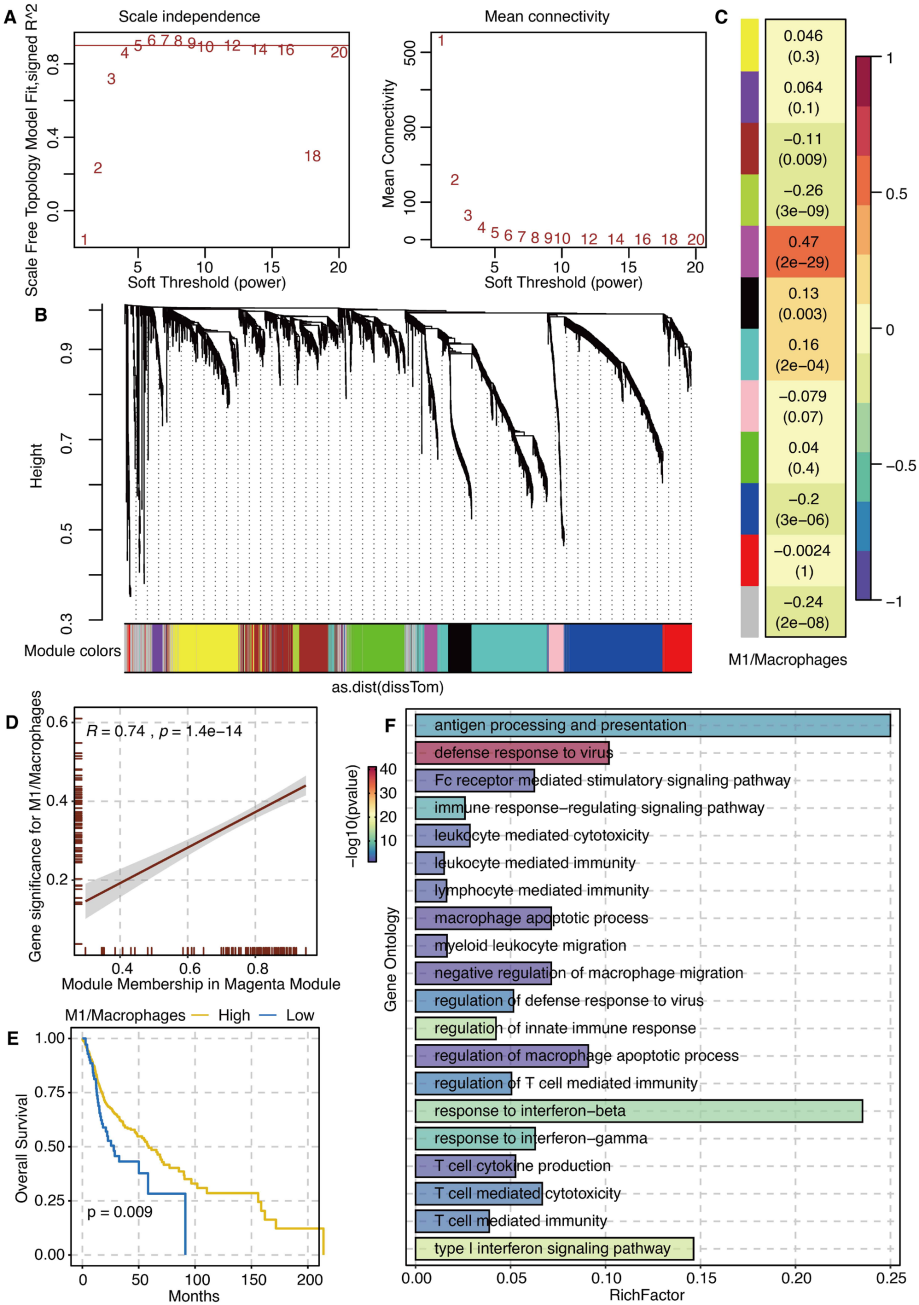
WGCNA on M1/Macrophage. (A) Scale independence and mean connectivity for WGCNA. (B) Cluster dendrogram generating gene module related to M1/Macrophage. (C) Module‐trait relationship related to M1/Macrophage. (D) The correlation between gene significance for M1/Macrophage and module membership in the magenta module. (E) Survival curves of M1/Macrophage related groups. (F) GO enrichment analysis for magenta module genes.

Scale independence and mean connectivity for WGCNA are shown in Figure 1A. The cluster dendrogram generating gene module related to M1/Macrophage is shown in Figure 1B. Module‐trait relationship related to M1/Macrophage showed that the magenta module had the highest correlation (Figure 1C). The correlation between gene significance for M1/Macrophage and module membership in the magenta module is shown in Figure 1D. Survival curves of M1/Macrophage‐related groups revealed that a high M1/Macrophage ratio was associated with better survival (Figure 1E). GO enrichment analysis for magenta module genes revealed that antigen processing, presentation, and response to IFNβ were highly enriched (Figure 1F).

### 3.2 Development of M.Cluster Based on Magenta Module Genes

**FIGURE 2:**
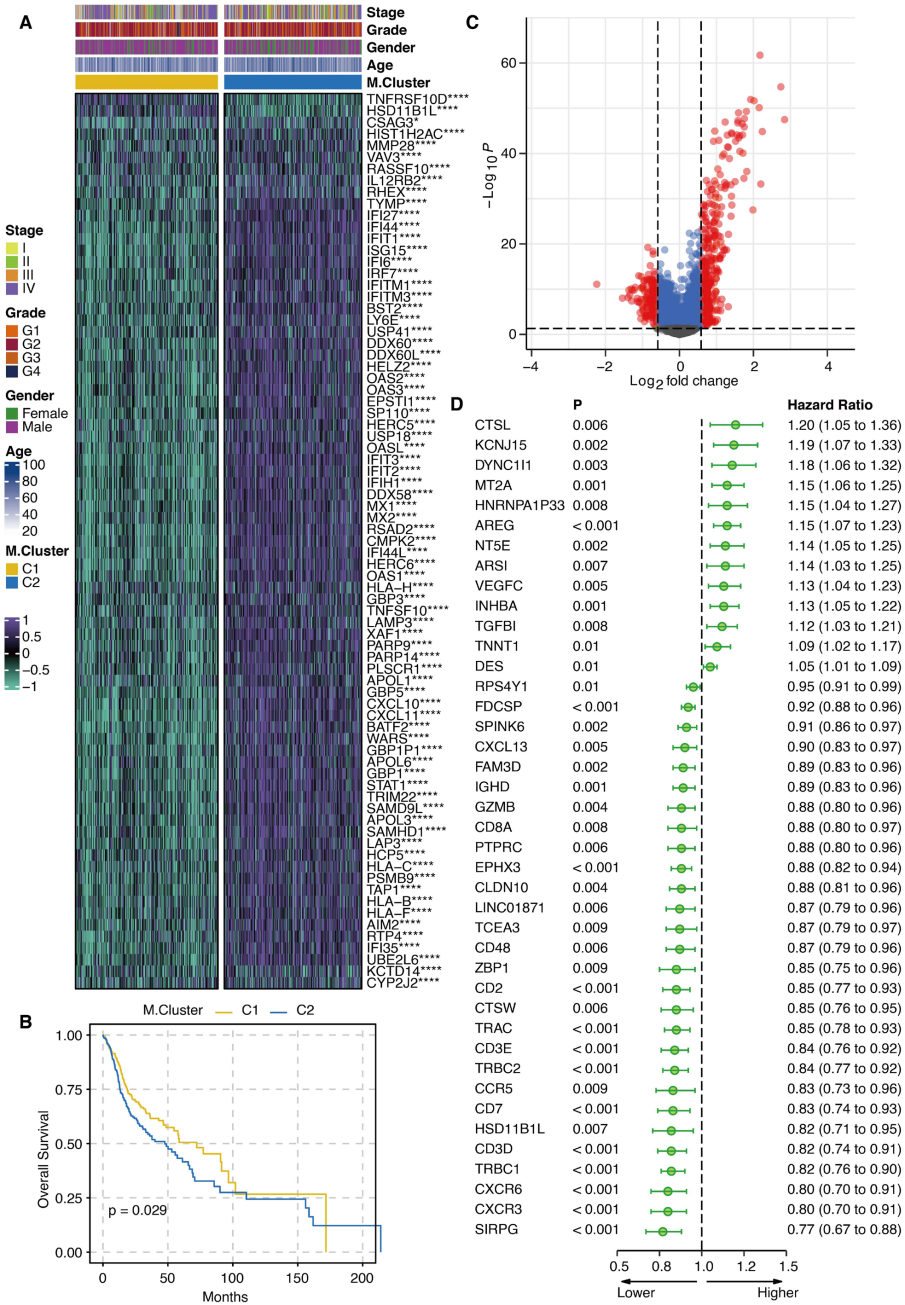
Development of M.Cluster based on magenta module genes. (A) Heatmap of the distribution of magenta module genes in M.Cluster. (B) Survival curves of M.Cluster. (C) Volcano plot of the DEGs in M.Cluster. (D) Univariate Cox regression analysis of the DEGs.

To further investigate the functional implications of the M1/Macrophage ratio‐related genes identified by WGCNA, we performed consensus clustering based on the expression of magenta module genes to classify patients into distinct molecular clusters, termed M.Cluster. This approach revealed two main subtypes, C1 and C2. Heatmap visualisation showed that most magenta module genes were highly expressed in the C2 M.Cluster (Figure 2A). Survival analysis indicated that patients in the C1 M.Cluster exhibited significantly better overall survival compared to those in C2 (Figure 2B). Volcano plot analysis highlighted differentially expressed genes (DEGs) between the two M.Cluster subgroups (Figure 2C). Subsequent univariate Cox regression analysis of these DEGs identified 13 genes as risk genes and 28 as protective genes (Figure 2D). To identify the most robust prognostic gene within the M.Cluster signature, we applied multiple machine learning algorithms for dimension reduction.

### 3.3 Machine Learning for Dimension Reduction of Prognostic DEGs

**FIGURE 3:**
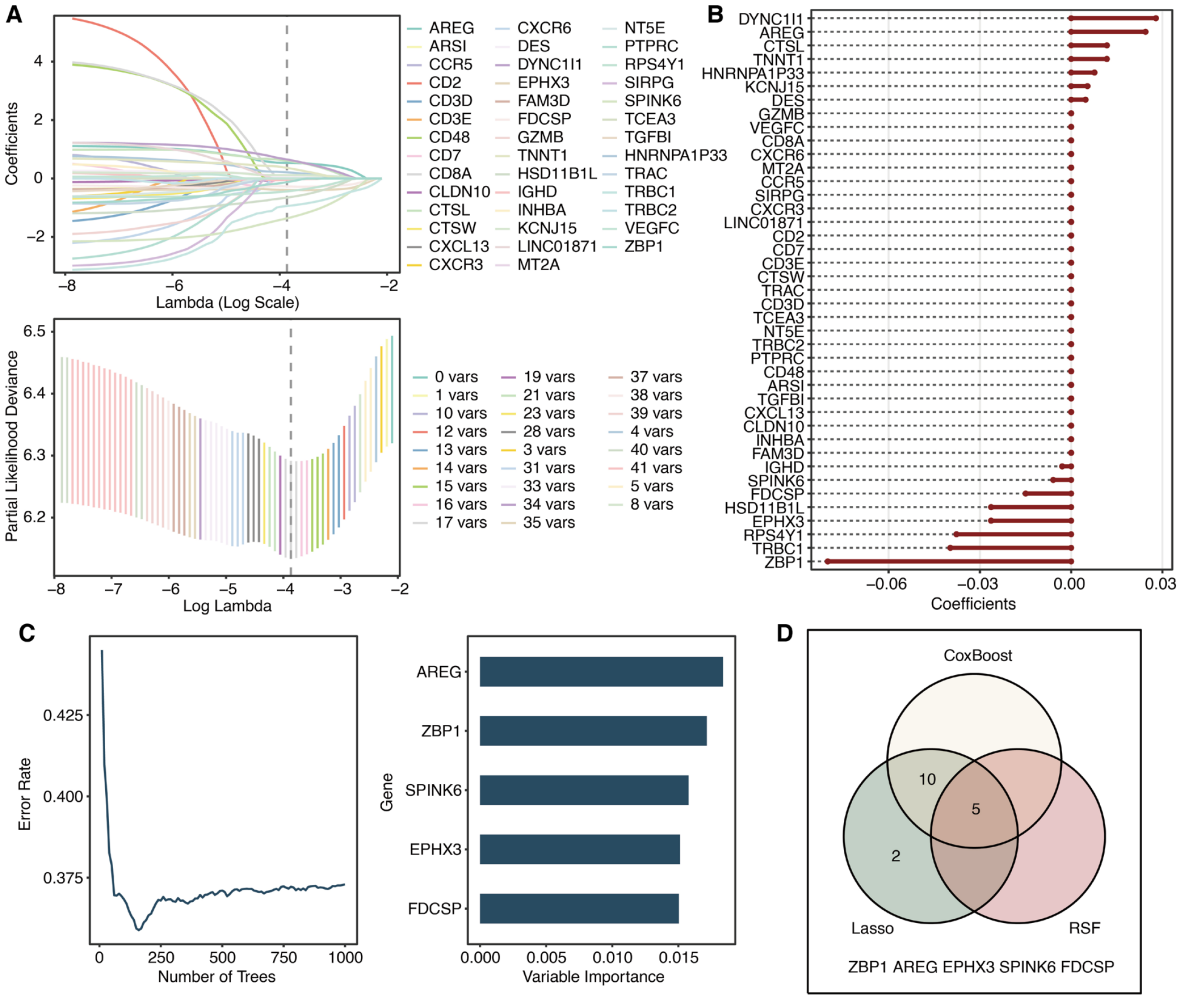
Machine learning for dimension reduction of prognostic DEGs. (A) LASSO regression analysis for dimension reduction of DEGs. (B) CoxBoost analysis for dimension reduction of DEGs. (C) RSF analysis for dimension reduction of DEGs. (D) Venn plot for the intersected genes.

To ensure robust and unbiased identification of the most prognostically significant genes from the DEGs, we employed three distinct machine learning algorithms, LASSO regression, CoxBoost, and random survival forest (RSF), each offering unique advantages in handling high‐dimensional survival data. The LASSO regression analysis selected a subset of features through L1 regularisation, as depicted in Figure 3A. The CoxBoost approach further refined the prognostic gene set by leveraging likelihood‐based boosting in the Cox model (Figure 3B), while the RSF algorithm accounted for non‐linear interactions and temporal effects in survival data (Figure 3C). A Venn diagram was used to identify the intersection of genes derived from the three methods, yielding five consensus genes with high prognostic reliability (Figure 3D). Among these, ZBP1 was prioritised for further experimental validation due to its well‐documented roles in immune regulation and necroptosis, as well as its consistent high ranking across all three machine learning models.

### 3.4 Functional Annotation of ZBP1

**FIGURE 4:**
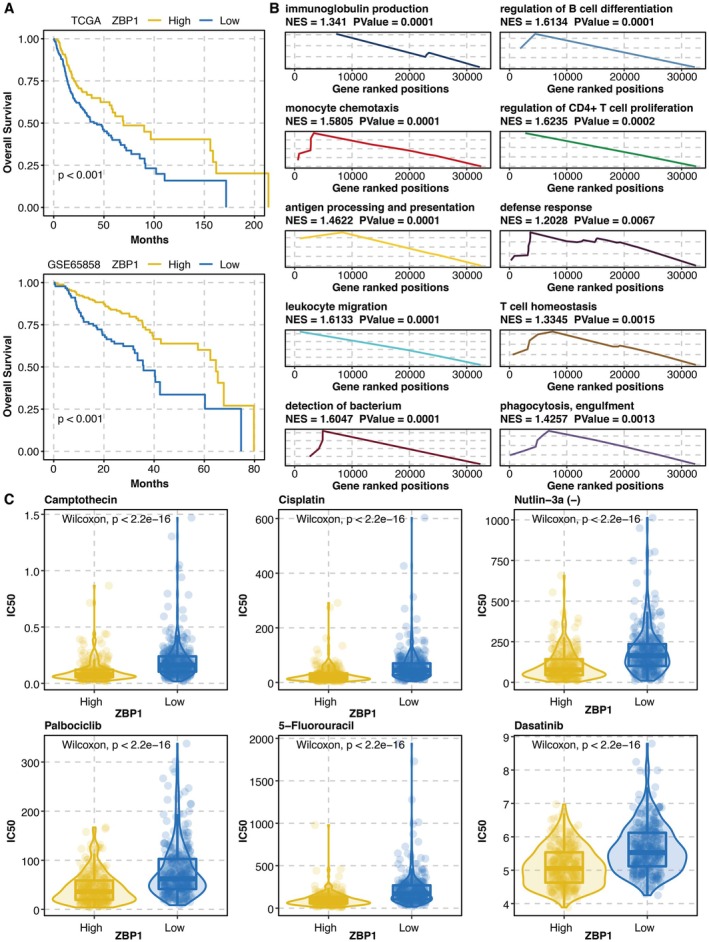
Functional annotation of ZBP1. (A) Survival curves of ZBP1‐related groups. (B) GSEA for ZBP1. (C) Drug prediction analysis for ZBP1.

Survival curves of ZBP1‐related groups revealed that high ZBP1 expression was associated with better survival (Figure 4A). GSEA for ZBP1 showed that immune activity, especially T cell activity, was highly enriched (Figure 4B). Drug prediction analysis for ZBP1 revealed that ZBP1 could predict IC50 of Camptothecin, Cisplatin, Nutlin‐3a (−), Palbociclib, 5‐Fluorouracil, and Dasatinib (Figure 4C).

### 3.5 Immune Characteristics of ZBP1

**FIGURE 5:**
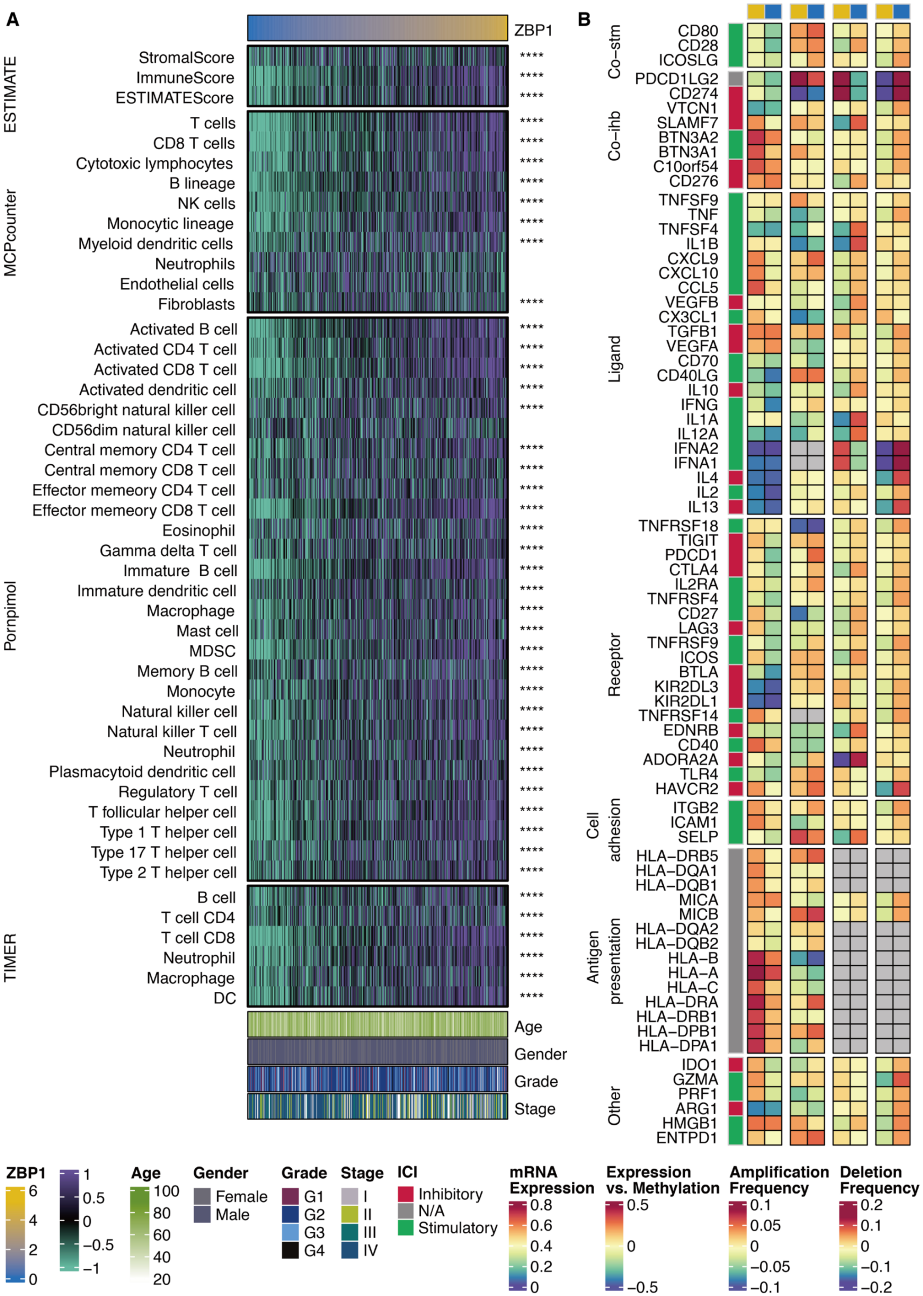
Immune characteristics of ZBP1. (A) Heatmap of the immune infiltrating cells related to ZBP1. (B) Heatmap of the immune modulators related to ZBP1.

Heatmap of the immune infiltrating cells related to ZBP1 showed that several immune cells, especially T cells and macrophages, were positively associated with ZBP1 (Figure 5A). Heatmap of the immune modulators related to ZBP1 showed that classical immune checkpoints, especially PD‐L1 and PD‐1, were positively associated with ZBP1 (Figure 5B).

### 3.6 Mutation Characteristics of ZBP1

Highly mutated genes in HNSCC samples expressing high ZBP1 are shown in Figure S1A, in which TP53, TTN, and CDKN2A were the top three mutated genes. Highly mutated genes in HNSCC samples expressing low ZBP1 are shown in Figure S1B, in which TP53, TTN, and FAT1 were the top three mutated genes.

### 3.7 In Vitro Validation on ZBP1

**FIGURE 6:**
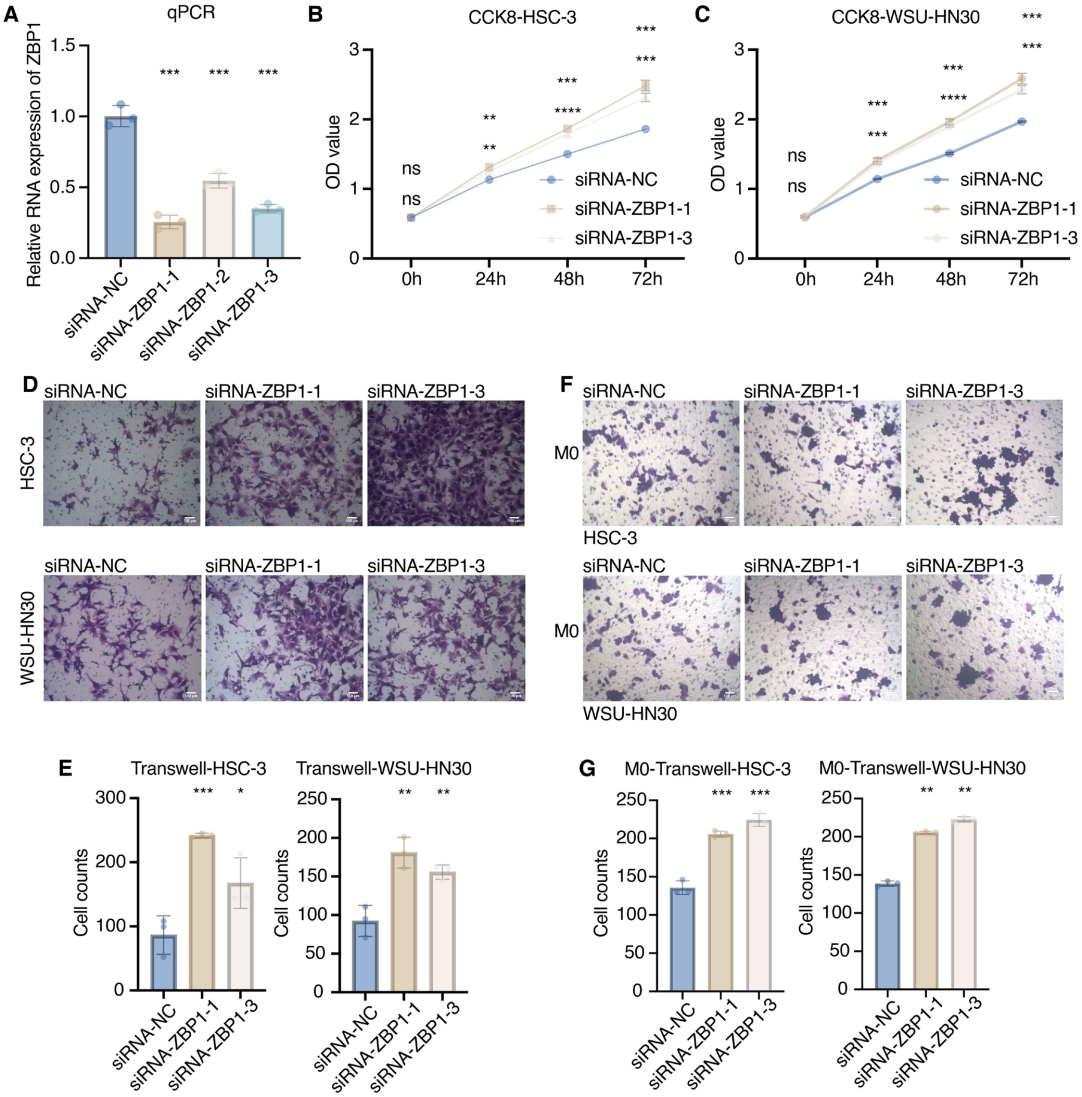
In vitro validation on ZBP1. (A) RT‐qPCR assay assessing the mRNA expression of ZBP1 in siRNA‐NC and three siRNA‐ZBP1 groups in HSC‐3 cells. (B) CCK8 assay assessing the proliferation ability of HSC‐3 cells. (C) CCK8 assay assessing the proliferation ability of WSU‐HN30 cells. (D) Transwell assay assessing the migration ability of HSC‐3 and WSU‐HN30 cells. (E) Statistical analysis of the Transwell assay. (F) Transwell assay assessing the migration ability of M0 macrophages after coculture with HSC‐3 and WSU‐HN30 cells. (G) Statistical analysis of the Transwell assay.

RT‐qPCR assay showed that three siRNA sequences could all effectively silence ZBP1 expression in HSC‐3 cells (Figure 6A). CCK8 assay showed that the proliferation ability of HSC‐3 and WSU‐HN30 cells was significantly promoted after silencing ZBP1 (Figure 6B,C). Transwell assay showed that the migration ability of HSC‐3 and WSU‐HN30 cells was significantly promoted after silencing ZBP1 (Figure 6D,E). Transwell assay showed that the migration ability of M0 macrophages after coculture with HSC‐3 and WSU‐HN30 cells was significantly promoted after silencing ZBP1 (Figure 6F,G).

## 4 Discussion

Macrophage polarisation is essential in cancer since it shapes the tumour microenvironment and influences tumour growth. In particular, macrophages exist in two primary polarisation states: M1 and M2 [24]. Because M1 macrophages can directly destroy cancer cells and stimulate an immune response against the tumour, they are pro‐inflammatory and anti‐tumorigenic. M2 macrophages, on the other hand, promote tissue healing and immunological suppression, which supports tumour growth, angiogenesis, and metastasis. As a result, they are anti‐inflammatory and pro‐tumorigenic. The ratio of M1 to M2 macrophages in the tumour microenvironment is crucial in regulating how cancer develops and how well a patient responds to treatment [25]. Comprehending the mechanisms governing macrophage polarisation in cancer may facilitate the creation of innovative immunotherapies and tailored therapeutic interventions [26]. M1/Macrophage ratio‐related genes from WGCNA were closely linked to M1 macrophages. In accordance, a high M1/Macrophage ratio predicted better survival in HNSCC. Besides, M1/Macrophage ratio‐related genes were highly enriched in immune activation pathways.

In clinical oncology, machine learning is increasingly utilised to detect cancer, forecast patient outcomes, and guide therapy decisions [27]. LASSO regression analysis, CoxBoost analysis, and RSF analysis were all machine learning algorithms handling high‐dimensional data and identifying essential features in understanding cancer progression and patient outcomes. In summary, LASSO regression analysis modifies the basic linear regression equation by incorporating a penalty factor. By reducing less significant variables to zero, this penalty term assists the model in including just the most pertinent variables. This enhances the predicted accuracy of the model and helps to avoid overfitting. CoxBoost aims to improve the model's predictive accuracy by iteratively fitting weak learners to the residuals of the Cox model. RSF uses an ensemble learning method that constructs many decision trees during training and outputs the class's mode or the individual trees' mean prediction. Therefore, the intersected gene, ZBP1, identified by three machine learning algorithms, was believed to be potent and reliable.

ZBP1, Z‐DNA binding protein 1, is a gene that encodes a protein that controls inflammation and immunological responses [28, 29, 30]. It contributes to the immune system's activation and identification of viral RNA. Numerous illnesses and ailments, such as viral infections and autoimmune disorders, have been connected to ZBP1 [31]. The functions and roles of ZBP1 in cancer have also been recently discovered. ADAR1 hides ZBP1‐driven necroptosis's potential as a cancer immunotherapeutic [32]. In breast cancer, glucose restriction induces ZBP1‐dependent necroptosis [33]. ADAR1 inhibits PANoptosis and ZBP1‐mediated immune response, which promotes carcinogenesis in melanoma and colorectal cancer [34]. In general, ZBP1 was a tumour suppressor. Consistent with the previous reports, high ZBP1 expression was associated with better survival in HNSCC. Besides, TP53, TTN, and CDKN2A were the top three mutated genes in HNSCC samples expressing high ZBP1. TP53 [35] and CDKN2A [36] were classical tumour suppressors. Besides, ZBP1 could suppress the proliferation and migration of HNSCC cells. Again, this finding proved the tumour‐suppressive role of ZBP1 in HNSCC.

It has been demonstrated that ZBP1‐activated innate immunity, which includes NF‐κB signalling and the type‐I interferon (IFN‐I) response, is a crucial line of defence against pathogenic infection. Furthermore, ZBP1‐mediated PANoptosis has conflicting effects on tumour immunity, auto‐inflammatory illnesses, and anti‐infection [37]. Notably, ZBP1 could activate T cell activity and B cell activity in HNSCC. Besides, ZBP1 could suppress M0 macrophage migration in HNSCC. Taken together, our findings proved the tumour‐suppressive role of ZBP1 in HNSCC.

Immunotherapy uses a patient's immune system to identify and eliminate tumour cells, including ICB, adoptive cell therapy, therapeutic vaccines, and oncolytic viruses. In recent years, immunotherapy has achieved remarkable results in treating many tumours, and immunotherapy research for HNSCC has also received wide attention [38, 39, 40]. ZBP1 was positively associated with several classical immune checkpoints, such as PD‐1, PD‐L1, TIGIT, and CD40. Therefore, ZBP1 could be a reliable immunotherapy determinant.

In this study, M1/Macrophage ratio‐related genes were identified by WGCNA and were further used as the input of consensus clustering for developing M.Cluster. M.Cluster could effectively discriminate patients' survival. Then, three machine learning algorithms, LASSO regression analysis, CoxBoost analysis, and RSF analysis, were used to screen out the most potent gene related to M.Cluster, ZBP1. ZBP1 was a powerful predictor of prognosis, immune activity, mutation, and immunotherapy. ZBP1 was expected to be explored further in vivo experiments and cohort validation. Clinically, ZBP1 expression could serve as a companion diagnostic to stratify patients for anti‐PD‐1/PD‐L1 immunotherapy, potentially enhancing response rates and personalising treatment strategies in HNSCC.

While previous studies have employed circadian genes [41], immune‐related signatures [42, 43], or HPV‐based classifiers [44] to stratify cancer risk, our work distinguishes itself by integrating macrophage polarisation dynamics with AI‐driven machine learning (LASSO‐CoxBoost‐RSF) for prognostic modelling in HNSCC. Unlike conventional approaches that rely on single‐omics or predefined gene sets, our M.Cluster framework leverages WGCNA‐derived M1/Macrophage ratio genes followed by rigorous feature selection, enabling robust identification of ZBP1 as a multimodal biomarker linked to survival, immune activation, and immunotherapy response. Notably, compared to angiogenesis‐centric predictors [45], our strategy uniquely captures the interplay between TAMs and tumour suppression, a dimension underexplored in existing HNSCC studies. The concordance of ZBP1's tumour‐suppressive role across three ML algorithms further underscores the reliability of our AI‐ML pipeline, offering a replicable paradigm for translational research in immuno‐oncology.

## Author Contributions

The study was created and planned by O.S. and F.G. S.W. and K.W. collected the data, and F.G. carried out the bioinformatic analysis and wrote the manuscript. The statistical analysis was carried out by S.W. and T.F. under O.S.'s supervision. The manuscript has been read, carefully amended, and approved by all authors.

## Conflicts of Interest

The authors declare no conflicts of interest.

## Supporting information


**Figure S1:** Mutation characteristics of ZBP1. (A) Highly mutated genes in HNSCC samples expressing high ZBP1. (B) Highly mutated genes in HNSCC samples expressing low ZBP1.

## Data Availability

Data analysed in this study was obtained from the TCGA‐HNSC project (https://portal.gdc.cancer.gov/) and GEO accession GSE65858 (https://www.ncbi.nlm.nih.gov/geo/query/acc.cgi?acc=GSE65858).
