# Supplementary material for: ZBP1, an M1 Macrophage‐Associated Biomarker Identified by Machine Learning, Suppresses Tumorigenesis and Predicts Immunotherapy Response in Head and Neck Squamous Cell Carcinoma
Source: J Cell Mol Med. 2025 Nov 26;29(22):e70953. doi: 10.1111/jcmm.70953 (PMC12657136; doi:10.1111/jcmm.70953)
Supplement: Supplementary file 1 — Figure S1: Mutation characteristics of ZBP1. (A) Highly mutated genes in HNSCC samples expressing high ZBP1. (B) Highly mutated genes in HNSCC samples expressing low ZBP1. [file JCMM-29-e70953-s001.docx]

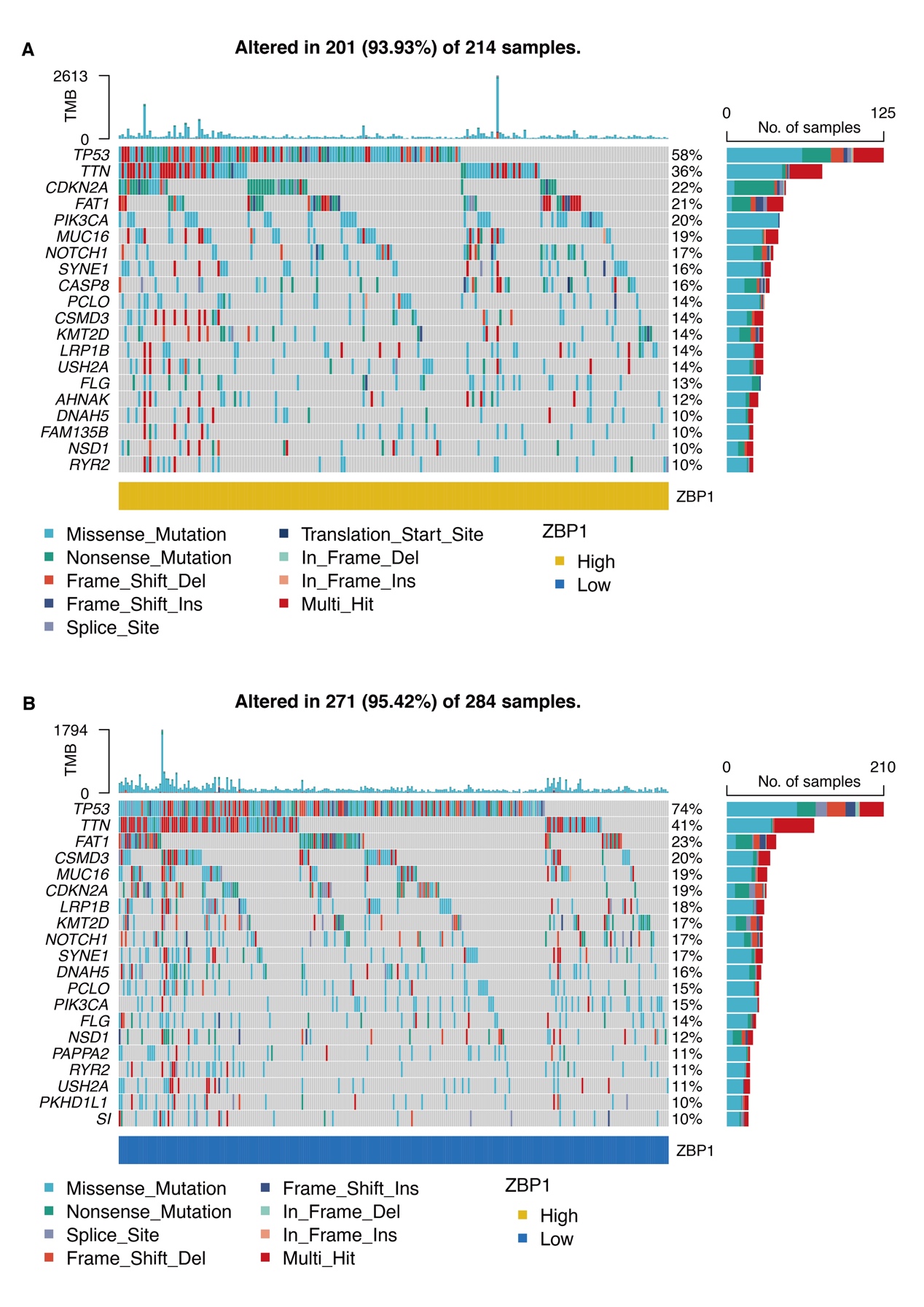


Figure S1. Mutation characteristics of ZBP1. A. Highly mutated genes in HNSCC samples expressing high ZBP1. B. Highly mutated genes in HNSCC samples expressing low ZBP1.
